# Non-Neuronal Cells Are Required to Mediate the Effects of Neuroinflammation: Results from a Neuron-Enriched Culture System

**DOI:** 10.1371/journal.pone.0147134

**Published:** 2016-01-20

**Authors:** Chin Wai Hui, Yang Zhang, Karl Herrup

**Affiliations:** Division of Life Science and the State Key Laboratory of Molecular Neuroscience, Hong Kong University of Science and Technology, Clear Water Bay, Kowloon, Hong Kong; University of South Florida, UNITED STATES

## Abstract

Chronic inflammation is associated with activated microglia and reactive astrocytes and plays an important role in the pathogenesis of neurodegenerative diseases such as Alzheimer’s. Both in vivo and in vitro studies have demonstrated that inflammatory cytokine responses to immune challenges contribute to neuronal death during neurodegeneration. In order to investigate the role of glial cells in this phenomenon, we developed a modified method to remove the non-neuronal cells in primary cultures of E16.5 mouse cortex. We modified previously reported methods as we found that a brief treatment with the thymidine analog, 5-fluorodeoxyuridine (FdU), is sufficient to substantially deplete dividing non-neuronal cells in primary cultures. Cell cycle and glial markers confirm the loss of ~99% of all microglia, astrocytes and oligodendrocyte precursor cells (OPCs). More importantly, under this milder treatment, the neurons suffered neither cell loss nor any morphological defects up to 2.5 weeks later; both pre- and post-synaptic markers were retained. Further, neurons in FdU-treated cultures remained responsive to excitotoxicity induced by glutamate application. The immunobiology of the FdU culture, however, was significantly changed. Compared with mixed culture, the protein levels of NFκB p65 and the gene expression of several cytokine receptors were altered. Individual cytokines or conditioned medium from β-amyloid-stimulated THP-1 cells that were, potent neurotoxins in normal, mixed cultures, were virtually inactive in the absence of glial cells. The results highlight the importance of our glial-depleted culture system and identifies and offer unexpected insights into the complexity of -brain neuroinflammation.

## Introduction

Primary neuronal culture is a simple and reliable system to study the behavior of neurons in isolation from both their normal cellular and chemical environment. Unlike most neuronal cell lines, mature primary neuronal cultures are postmitotic (in G0 phase) and are able to form stable functional synapses. As a result, these cultures allow us to study the neurobiology of different brain regions in isolation.

Embryonic neural precursor cells are able to differentiate into glial cells and neurons [1]; therefore, most cultures of embryonic brain represent a mixture of glial cells, neurons, innate immune system cells and fibroblasts. Culture media have been developed that favor the survival of neurons [2]; nonetheless with increasing time in culture, the mitotic, non-neuronal cell populations tend to increase their representation. This reduces the precision of attempts to accurately define the cellular nature of any of a myriad complex responses—electrophysiological, immunological or molecular.

Previous studies have shown that anti-mitotic agents, namely arabinosylcytosine C (AraC) and 5-Fluoro-2’-deoxyuridine (FdU), remove proliferating glial cells and fibroblasts but maintain neurons in primary cultures [3–10]. While this approach successfully eliminates all dividing cells in the short term, over longer culture periods, researchers have reported problems. Ahlemeyer et al. (2003) have shown that AraC unexpectedly activates astrocytes resulting in damage to neurons during glutamate excitotoxicity [11]. Direct effects of the agents are also reported, specifically, evidence that AraC kills postmitotic neurons by a mechanism similar to neurotrophic factor deprivation. Enhanced DNA damage was also reported at the concentrations used in previously reported purification methods [12, 13]. Images from Zhou et al. (2012) demonstrate that neurons in AraC treated cultures appear unhealthy when compared to untreated cultures [14]. These findings suggest that chronic in vitro use of AraC may adversely change neuronal features and affect neuronal function and possibly fate.

In the current study, we have modified earlier protocols in order to isolate the neuronal response to an immune system challenge. We used two-week cortical neuronal cultures exposed to a newly developed transient FdU treatment regime to eliminate most non-neuronal cells. The treatment is especially useful as neuronal loss is minimal and their healthy appearance is maintained, even while ~99% of the proliferating, non-neuronal cells are lost. Under these conditions, we demonstrate that the presence of glial cells is required to trigger an inflammation-induced neurodegeneration. The findings highlight the importance of our modified culture system and have significance for understanding the pathways by which neuroinflammatory events bring damage to the cells of the CNS.

## Methods and Materials

### Animals

All animals were housed at the accredited Animal and Plant Care Facility of Hong Kong University of Science and Technology (HKUST). All animal work was approved by the HKUST Institutional Animal Care and Use Committee and was in full accordance with all Hong Kong Department of Health guidelines. Animal sacrifice is a necessary part of this work, as we use primary cortical cells in our experiments. The rapid harvesting of living neural tissue requires the use of cervical dislocation of unanaesthetized females as anesthesia would interfere with the health and function of the isolated brain cells. Tissue for cortical neuron culture was harvested from the cerebral cortex of E16.5 embryos. The embryos were removed from pregnant dams generated by mating C57BL/6 males and females for one night. The following morning (embryonic day 0.5 –E0.5) the females were separated and observed for successful pregnancies. On E16.5, the gravid female was restrained on a flat surface by grasping the tail. With a single movement the skull is pinned to the surface while opposing traction is applied to the tail until the cervical spinal column separates. Death is nearly instantaneous so that little distress or discomfort is experienced.

### Primary cortical neuronal and neuroblastoma cultures

Embryonic cortical neurons were isolated by standard procedures. E16.5 embryonic cerebral cortices were treated with 0.25% Trypsin-EDTA and dissociated into single cells by gentle trituration. Cells were suspended in Neurobasal medium supplemented with B27 and 2 mM GlutaMAX, then plated on coverslips or dishes coated with poly-L-Lysine (0.05 mg/mL) diluted in boric buffer. Enrichment of neuronal culture was performed using both a previously reported method and a modified protocol. For the previously reported method [5], 5 μM 5-fluoro-2'-deoxyuridine (FdU) from Sigma (F0503, St. Louis, MO, USA) was added into the culture at DIV4. Cultures were then replenished by replacing half of the volume of medium, but still containing 5 μM FdU, every 3–4 days until DIV14. For our modified protocol, 1 or 2 μM FdU added at DIV4 and incubated for 24hr to kill the proliferating cells. Medium containing FdU was then replaced with fresh medium without FdU (old to new NB medium in 1:1 ratio). Half of the medium was then replaced every 5 days until at least 14 days in vitro (DIV) before any treatment.

Murine neuroblastoma N2a cells were cultured in standard DMEM media supplemented with 10% FBS for routine passage. N2a cells and primary cortical neurons were treated with lipopolysaccharide (LPS, 1 μg/ml) (L2880, Sigma), tumor necrosis factor alpha (TNFα, 100 ng/ml) (#1050, BioVision, Milpitas, California, USA) or interleukin 1 beta (IL1β, 50ng/ml) (#4128, BioVision) for 1 and 24 hours. Celastrol (Sigma) was added to the cultures for 24 hours to study NFκB signaling pathways. For histological study, cells were washed with PBS and fixed in 4% PFA for 15 minutes. After rinsing in PBS, cells were immersed in 0.1% PFA for long-term storage.

### THP-1 cell culture and conditioned medium

Human monocyte THP-1 cells were cultured in RPMI 1640 medium with 10% FBS and 0.05 mM β-mercaptoethanol for routine passage. The procedures of conditioned medium preparation have been described [15]. In brief 36 μl of 10 μM β-amyloid_1-42_ (#1428, Tocris, Bristol, United Kingdom) was added to 6-well plates and allowed to air dry on the surface of the wells. Following this, the wells were sterilized by exposure to UV light for 15 min. To make Aβ stimulated THP-1 conditioned medium (AM), THP-1 cells were plated in the wells coated with Aβ at a density of 180,000 per well in Neurobasal medium. After incubation for 2 days, the medium was collected, cleared of debris and loose cells by centrifugation, and used to treat neuronal cultures.

### EdU proliferation assay

5-ethynyl-2 ´-deoxyuridine (EdU) was used as a tool to monitor cell cycle reentry in cortical neuron. 10 μM EdU was added to the cell culture for 24h for incorporation into the genome of cells undergoing DNA replication. EdU labeling was performed according to the manufacturer’s protocol of Click-iT EdU cell proliferation assay kit Life Technologies (Grand Island, NY, USA). After EdU labeling, the samples were processed for immunofluorescence or DAPI labeling before mounting.

### Antibodies for histological and Western studies

PCNA, NFκB p105/50, NFκB p65 and phospho-NFκB p65 (Ser536) antisera were purchased from Cell Signaling Technology (Danvers, MA, USA); PSD95, GFAP, Ki67, MAP2, NFκB p105/50, NFκB p65 and GAPDH antisera were purchased from Abcam (Cambridge, MA, USA); c-Rel and actin antisera were purchased from Santa Cruz Biotechnology (Dallas, Texas, USA); Synapsin-I, Homer-1 and NG2 antisera were purchased from Millipore (Billerica, MA, USA). Secondary antisera conjugated with fluorescent Alexa dye 488 and 647 and Cy3 were purchased from Invitrogen and Jackson Laboratories. HRP-conjugated secondary antibodies were purchased from Cell Signaling Technology or Invitrogen.

### Immunofluorescence

Immunofluorescence was performed according to standard methods. Cells were blocked in 5% donkey serum diluted in PBS containing 0.3% Triton X-100 for 1 hour at room temperature and incubated with primary antibodies overnight. After rinsing in PBS, they were incubated for 1 hour at room temperature with secondary antibodies. Cells were then rinsed in PBS and counter-stained with DAPI for 3 minutes at room temperature. After rinsing, all coverslips were mounted with anti-fading hard-set fluorescence media from Vector Laboratories (Burlingame, CA, USA) on glass slides.

### Cell counting

For isolated primary cortical neurons, five fields were randomly chosen for quantification using a 20x objective on an Olympus fluorescent microscope. The percentage of positive cells with markers of interest was counted along with the total number of cells labeled with MAP2 (neurons) as well as the total number of DAPI-positive cells.

### Western Blot analysis

Cortical neuronal cultures were homogenized in RIPA lysis buffer (Millipore) containing protease and phosphatase inhibitors (Roche, Grenzacherstrasse, Basel, Switzerland). Tissue or cell lysates were centrifuged at 15,000 rpm at 4°C for 20 minutes. Protein levels in the supernatant were determined by Protein Assay solution (Bio-Rad, Hercules, CA, USA). Protein samples were diluted with 4x loading buffer (composition: 0.02% bromophenol blue, 40% glycerol, 250 mM Tris-HCl, 5% β-mercaptoethanol) and RIPA lysis buffer to the required concentration then denatured at 95°C for 5 minutes. Protein samples were separated by gel electrophoresis and transferred to nitrocellulose membrane (Bio-Rad). After blocking with TBST containing 5% bovine serum albumin (BSA, Sigma) or 5% milk (Bio-Rad), membranes were incubated in primary antisera overnight. After rinsing in TBST, they were incubated in HRP-conjugated secondary antibodies at room temperature for 1 hour. Membranes were washed in TBST and protein signals were visualized using ECL substrate reagents from Thermo-scientific (Waltham, MA, USA). The intensities of the bands were quantified by ImageJ and normalized with actin or GAPDH level.

### Reverse transcription and quantitative PCR

Cortical neuronal cultures were homogenized in lysis buffer containing 1% β-mercapethanol and total RNA was extracted using PureLink RNA mini-kit (Life Technologies) according to the manufacturer’s protocol. Subsequently, same amount of total RNA was reverse transcribed into total cDNA using PrimeScript II first strand cDNA synthesis kit from Takara Biotechnology (Otsu, Shiga, Japan). cDNA was diluted to suitable concentration before use. Real-time PCR was performed using SYBR Premix Ex Taq (Takara Biotechnology) in 7500 Real-Time PCR System (Applied Biosystems, Life Technologies). ROX II was applied as the reference dye. The sets of primers used are listed in Tables 1 and 2; the expression level of *Gapdh* was used for normalization.

**Table 1:** Primer sequences for Homo sapiens.

Gene	Forward primer (5’->3’)	Reverse primer (5’->3’)
*18sRNA*	TGCATGTCTAAGTACGCACGGCC	GATAGGGCAGACGTTCGAATGGG
*TNFα*	TCCTTCAGACACCCTCAACC	AGGCCCCAGTTTGAATTCTT
*IL1β*	CAGCCAATCTTCATTGCTCA	GCATCTTCCTCAGCTTGTCC
*IL6*	AAAGAGGCACTGGCAGAAAA	CAGGGGTGGTTATTGCATCT
*IL8*	GGTGCAGTTTTGCCAAGGAG	TTCCTTGGGGTCCAGACAGA
*IL10*	TGGTGAAACCCCGTCTCTAC	TGGTGAAACCCCGTCTCTAC
*IFNG*	CCAACGCAAAGCAATACATGA	CCTTTTTCGCTTCCCTGTTTTA

**Table 2:** Primer sequences for Mus musculus.

Tnfrsf1α	CAGTCTGCAGGGAGTGTGAA	CACGCACTGGAAGTGTGTCT
Tnfrsf1β	AGATCCCAACCCCTGGATCA	GAGGCACCTTGGCATCTCT
Tgfβr1	GGCGAAGGCATTACAGTGTTT	TGAAAAAGGTCCTGTAGTTGGG
Tgfβr2	GCAAGTTTTGCGATGTGAGA	GGCATCTTCCAGAGTGAAGC
Tgfβr3	AATCCCAAATGGAGGTTTCC	GGCTCTCTGTGGTCTGGAAG
Il1r1	TAAGTAATGCTGTCCTGGGCTG	CAAATGAGCCCCAGTAGCAC
Il1r2	GAGGGGCTACACCACCAGTA	GGATTCGAGGCAACACATTT
Ifnγr1	GTAGCCTCACCGCCTATCAC	GGGCCTCTCCTGTGAGTCTA
Il6rα	GCACTCCTTGGATAGCAGAG	TGGACGAGGATTCTTGCACT
Il8rα	AGCTGGTGCCTCAGATCAAA	ATCACCAGCGAGTTTCCCAG
Il8rβ	ACTGCCTCCTACCCATCAGAA	TTCCTGTGTGAGGGGACTCTG
Il10ra	TCATGGTGACATTCCAGGGC	TGGAGGCCAAGCCAAATCAT
Il10rβ	CTTCCTTCTGGTGCCAGCTCTA	AGCCCTGACTCTCACAGTGTA
Tlr4	TGGCTGGTTTACACGTCCAT	TGCAGAAACATTCGCCAAGC
Gapdh	GGAGAAACCTGCCAAGTATGA	GGTCCTCAGTGTAGCCCAAG

### Statistical analysis

One-way ANOVA or unpaired Student’s *t-test* (Prism, GraphPad software, Version 5) were used to determine the differences in values between different groups. *p* < 0.05 was considered statistically significant. Abbreviation: */#: *p*< 0.05, **/##: *p*< 0.01, ***/###: *p*< 0.001.

## Results

### Optimal concentrations of FdU for the elimination of non-neuronal cells

We have previously shown that the immune response plays an important role in the neuronal cell death associated with neurodegenerative diseases such as Alzheimer’s disease and ataxia telangiectasia [16–18]. Unanswered by these earlier studies were questions concerning whether the effects of inflammation on neurons were direct events or indirect effects requiring the participation of other cells types. To pursue this question, we sought to develop protocols to establish a healthy, pure neuronal culture.

As mature neurons are post-mitotic, previous authors have used a variety of mitotic poisons to kill the mitotically active, non-neuronal cells. Most of these protocols involve the continuous presence of 5–10 μM FdU beginning at DIV4 of culture. We replicated these studies and, as an alternative, developed what we hoped would be a less harsh alternative. We reasoned that if the non-neuronal cells in our culture were continuously dividing, then we need only expose our cultures for a period long enough to cover a single non-neuronal cell cycle, thereby killing what would be in effect the progenitors of a potentially expanding non-neuronal population. In this modified protocol, FdU was added for only 24 hours at DIV4. After this, we returned the cultures to normal Neurobasal medium without FdU and continued growth.

**Fig 1:**
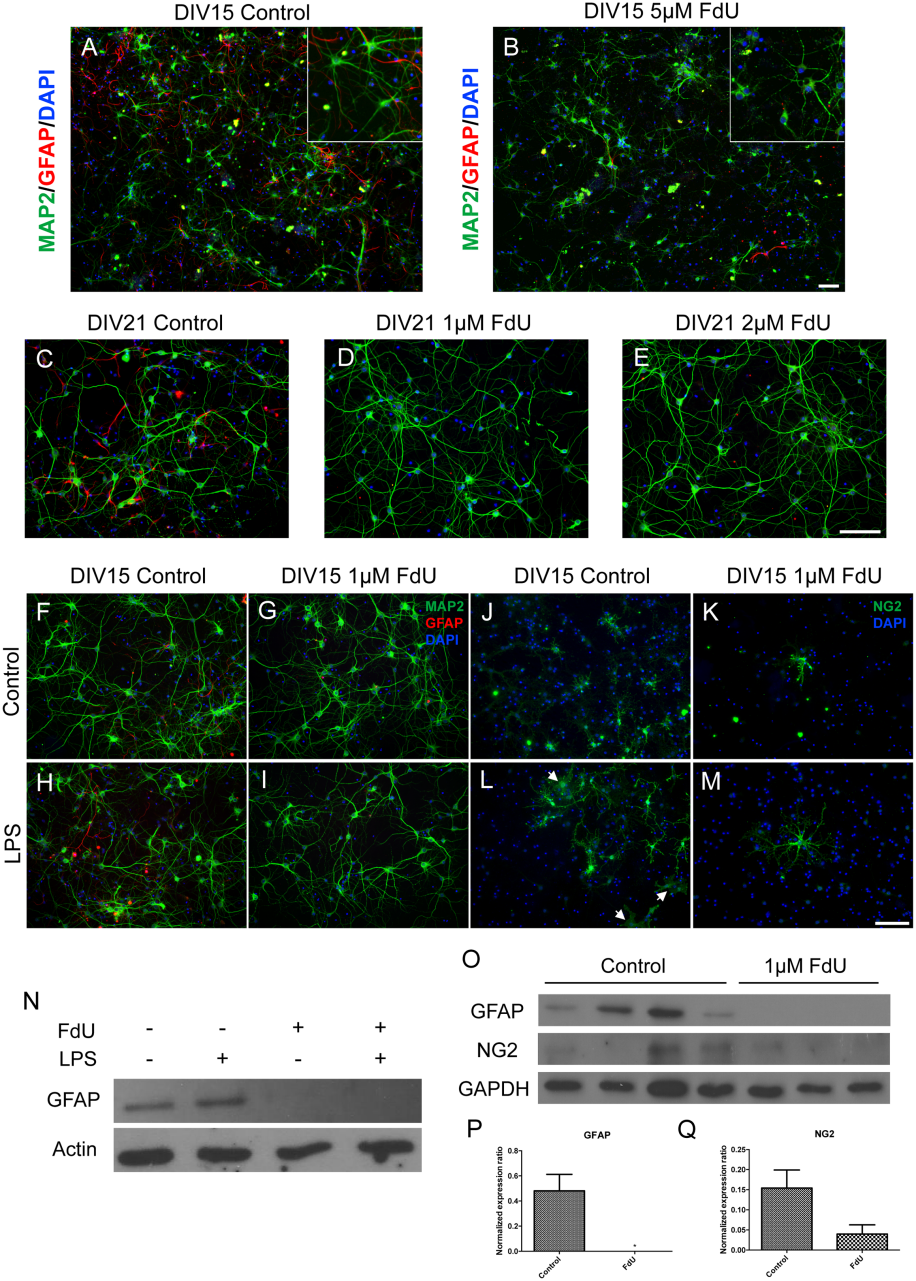
FdU depletion of non neuronal cells. (A) Control culture of mouse embryonic cortical neurons stained for neurons (Map2, green), astrocytes (GFAP, red) and DAPI (blue). (B) Sister culture of A treated with 5 μM FdU shows reduced astrocyte presence but also damaged neurons. (C-D) Low concentration FdU treatment (C) Control, (D) 1 μM, (E) 2 μM FdU applied at DIV 4 for 24hr followed by growth until DIV 15. (F-M) No evidence for quiescent non-neuronal cells. Control (F,H,J,L) or FdU treated (G,I,K,M) cortical cultures were stimulated with LPS (H,I,L,M) then stained for the GFAP (F-I) or NG2 (J-M). The white arrows in Panel L illustrate NG2-positive astrocyte-like cells that appear in LPS-treated control cultures. Scale bar = 50μm. *: *p*< 0.05. n = 4 for mixed culture and n = 3 for FdU culture. (N) Western blot of GFAP levels in normal (Lanes 1,2) or FdU-treated (Lanes 3,4) cultures after LPS treatment. Actin served as a loading control. (O) Western blots of GFAP or NG2 in control (Lanes 1–4) and FdU treated (Lanes 5–7) cortical neuron cultures. (P) Quantification of the levels of GFAP (normalized to the levels of actin). (Q) Quantification of the normalized levels of NGS.

Both the original and our modified protocol greatly reduced the number of GFAP-positive cells in the cultures (Fig 1A, 1B, 1F and 1G). The original protocol, however,also led to significantly reduced neuronal counts (Fig 1B). In our short-exposure protocol, not only were neuronal numbers unaffected, the surviving neurons maintained their robust morphological appearance at DIV14 (Fig 1F and 1G), as well as DIV21 (Fig 1C–1E)– 10 and 17 days after treatment respectively. Even after the 2½ week recovery times GFAP-positive cells were nearly undetectable. The absence of GFAP in our cultures was confirmed by Western Blot (Fig 1O and 1P). Another type of glial cell, microglia, was virtually absent in our mixed and FdU cultures (data not shown).

**Fig 2:**
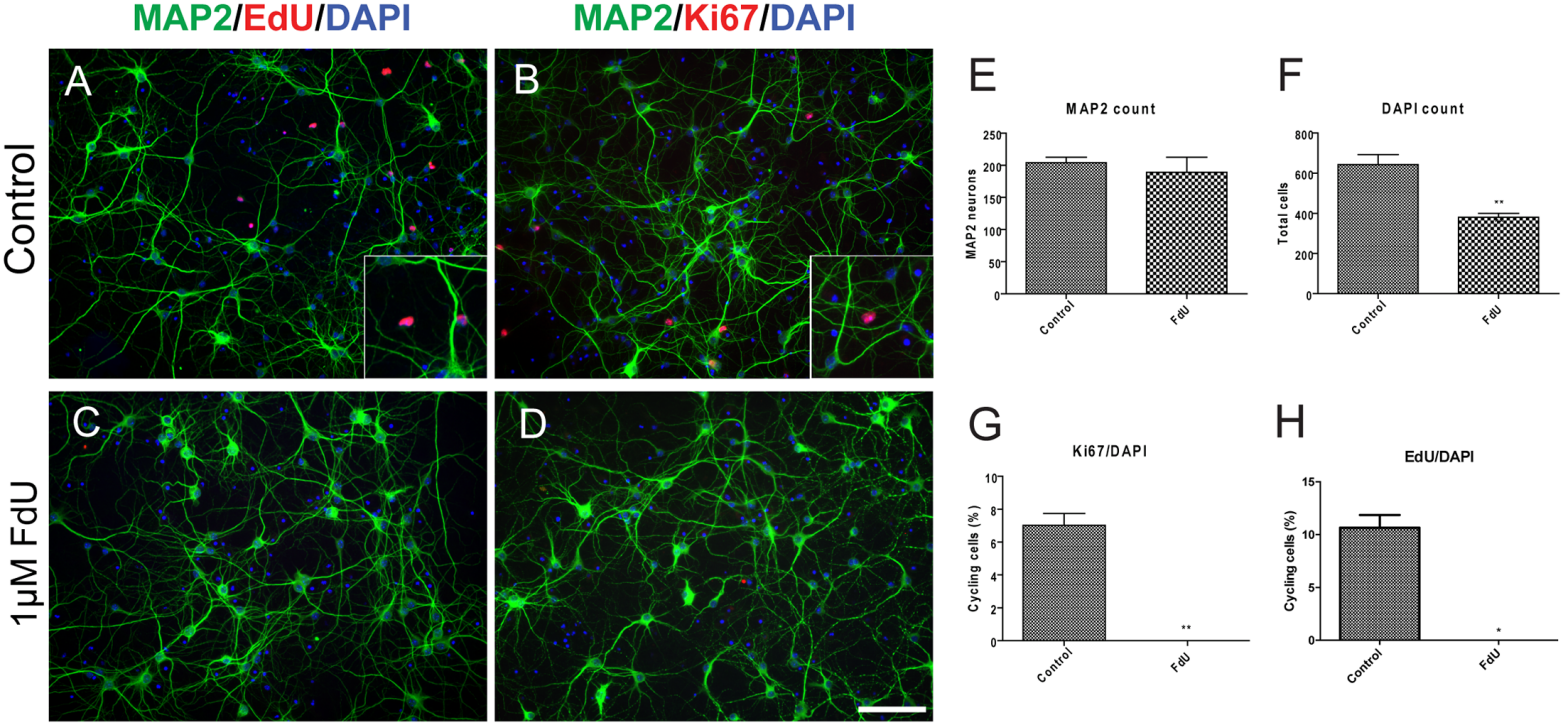
FdU killed proliferating cells while maintained neuronal survival. Cell cycle activity in neurons (Map2-positive cells) was monitored either with EdU (A,C) or Ki67 (B,D) in control (A,B) or FdU-treated (C,D) cells. (E-H) Quantification of the number of double labeled cells. Scale bar = 50μm. *: *p*< 0.05, **: *p*< 0.01. n = 4 for mixed culture and n = 3 for FdU culture.

We validated our model of FdU-induced precursor cell destruction by examining the cultures for continuing mitotic events. The short-exposure FdU treatment, as expected, eliminated all detectable cell cycle activity (Ki67 immunostaining) and DNA replication (EdU incorporation) in the cells remaining in DIV14 cultures (Fig 2A–2D, 2G and 2H). As predicted, from these findings, the FdU treatment reduced total DAPI counts at DIV14 while MAP2 counts remained unchanged (Fig 2E and 2F). This is consistent with the results in Fig 1 and argues that dividing non-neuronal cells were largely eliminated.

To test whether short term FdU treatment left behind cryptic glial precursor cells, we treated the FdU short-exposure cultures with bacterial lipopolysaccharide (LPS) as an inflammatory stimulus. LPS signals primarily through Toll-like receptor 4 (TLR4), which can be found on the surface of microglia (Iba-1-positive), astrocytes (GFAP-positive) and oligodendrocyte precursor cells (NG2-positive) [19, 20]. In FdU-untreated (mixed) cultures, LPS induced robust astrocyte activation (Fig 1H). In FdU-treated cultures, by contrast GFAP immunopositive cells were undetectable (Fig 1I). GFAP Western blots confirmed this finding (Fig 1N). These results make it unlikely that there were invisible or cryptic astrocytic cells in the culture that could be activated.

NG2-positive oligodendrocyte progenitor cells (OPCs) are also present in untreated DIV15 cultures. In short-exposure FdU-treated cultures by contrast, few remained visible by immunocytochemistry at DIV15 cultures (compare Fig 1J with 1K). Unlike the near total elimination of astrocytes, however, a small number of NG2 cells appeared to survive the FdU treatment. Similar results were found on NG2 Western blots of DIV 15 cultures (Fig 1O and 1Q). Interestingly, in both types of cultures, LPS stimulation resulted in two types of OPC differentiation. The first resembled a flat fibroblastoid type cells (Fig 1L, white arrows); these were found only in untreated (mixed) cultures. The second type resembled the parent NG2 cell, but with a greatly expanded set of processes. This expanded NG2 morphology was found in both FdU-treated (Fig 1M) and untreated cultures. Thus, a small number of OPCs remain after the short-exposure FdU treatment, but these retain only a limited responsiveness to TLR4 stimulation.

### Synaptic density is unaffected after FdU treatment

**Fig 3:**
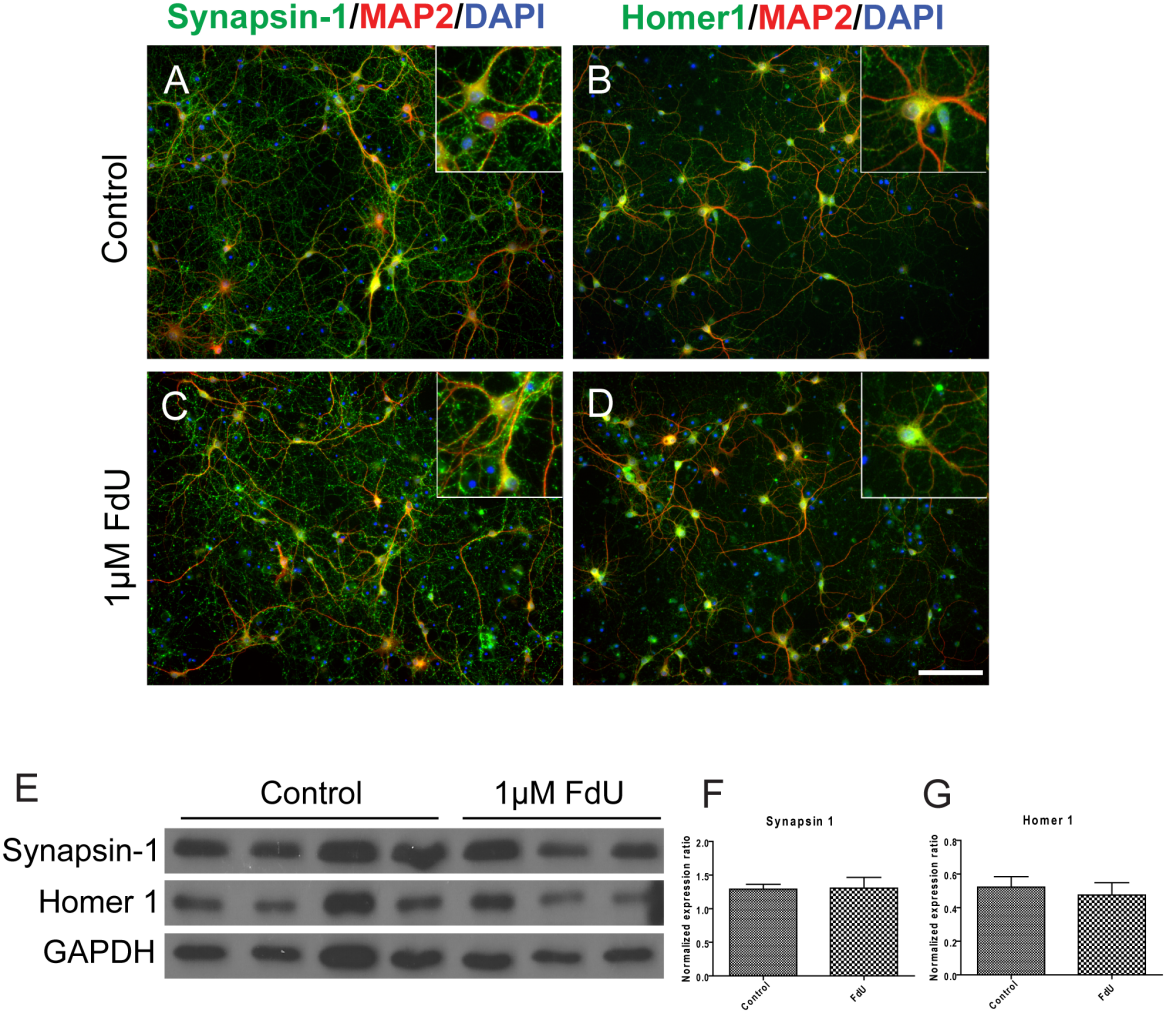
FdU treatment does not significantly diminish synaptic density. Synaptic structures in mixed (A,B) or FdU-treated (C,D) cultures were immunostained with pre-synaptic (synapsin-I, A,C) or postsynaptic (Homer-1, B,D) markers. Insets illustrate individual neurons stained with the indicated synaptic markers. Scale bar = 50 μm. n = 4 for mixed cultures and n = 3 for FdU cultures. (E-G) Protein expression was monitored by Western blot of different pre- (synapsin-I) and post- (Homer1) synaptic markers. (F,H) Quantification of the Western blots normalized to the actin loading control.

**Fig 4:**
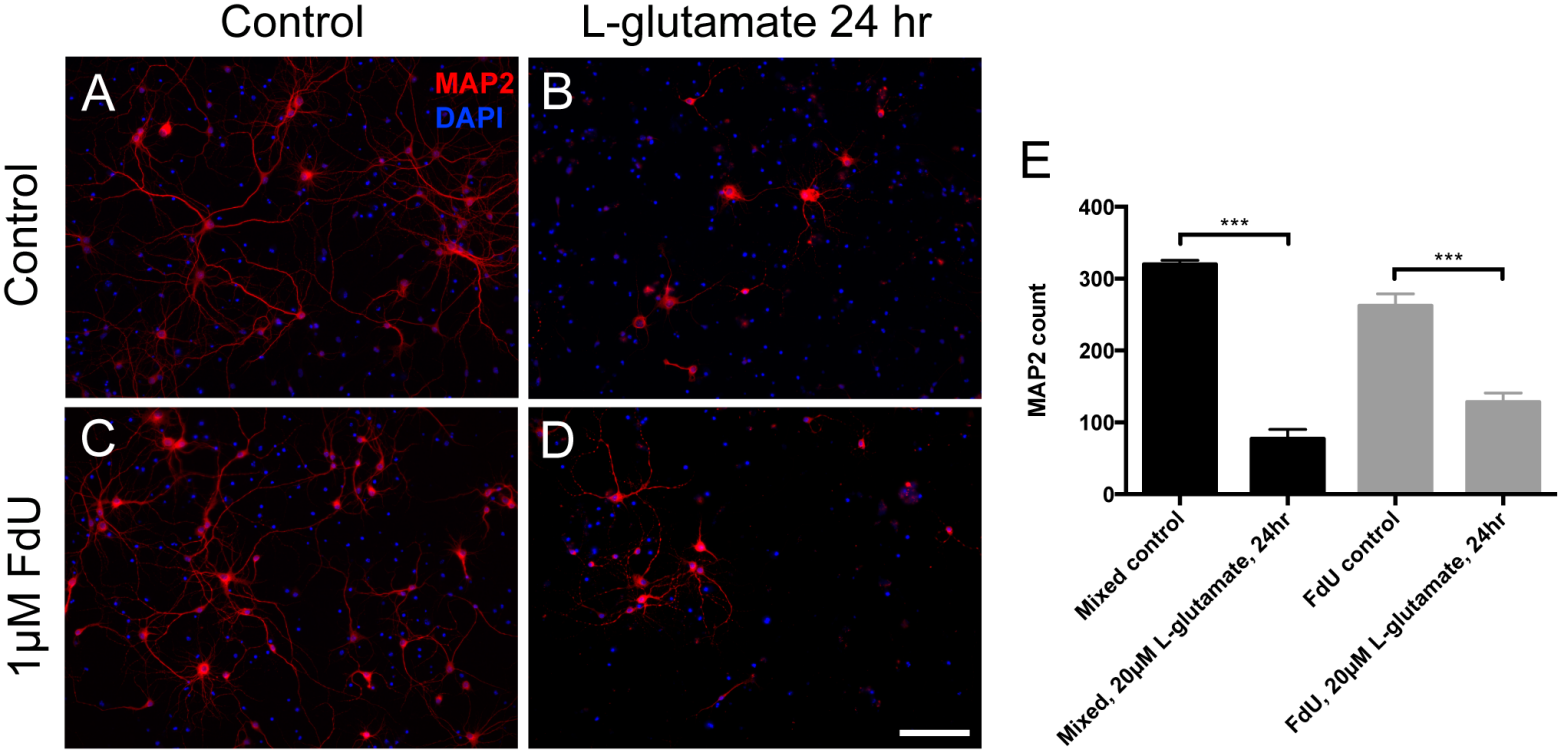
L-glutamate excitotoxicity is retained in the FdU culture. Both mixed (A, B) and FdU-treated (C, D) cultures showed loss of MAP2 positive cells after 24hr L-glutamate treatment. (E) Quantification confirmed the qualitative results. Scale bar = 50μm. ***: *p*< 0.001. n = 3 for each group.

To determine whether the FdU-exposed neurons maintained functionality, we first checked the synaptic density in the FdU cultures with different pre- and postsynaptic markers. There was no obvious loss of staining for synaptic structures as determined with synapsin-I (presynaptic, Fig 3A and 3C), Homer 1 (postsynaptic, Fig 3B and 3D) or PSD95 (postsynaptic, not shown). Western blot analysis of protein expression confirmed the immunocytochemistry (Fig 3E–3G). To test whether synaptic function was retained in FdU-treated cultures, we applied an excitotoxic insult– 20 μM L-glutamate [21, 22]. As would be predicted by the punctate pattern of appearance of postsynaptic markers, L-glutamate treatment led to comparable losses of MAP2-positive cells in both treated and untreated DIV14 cultures (Fig 4). In these experiments the neuronal number in the FdU culture trended lower from that in mixed cultures. even while they still maintained a healthy morphology and synaptic density. We also note that the percentage of cells lost was lower in the FdU-treated cultures, though not significantly so. This may suggest that a modest functional deficit is present despite the basic structural integrity.

### Maintenance of NFκB subunits and cytokine receptors in pure neuronal culture

**Fig 5:**
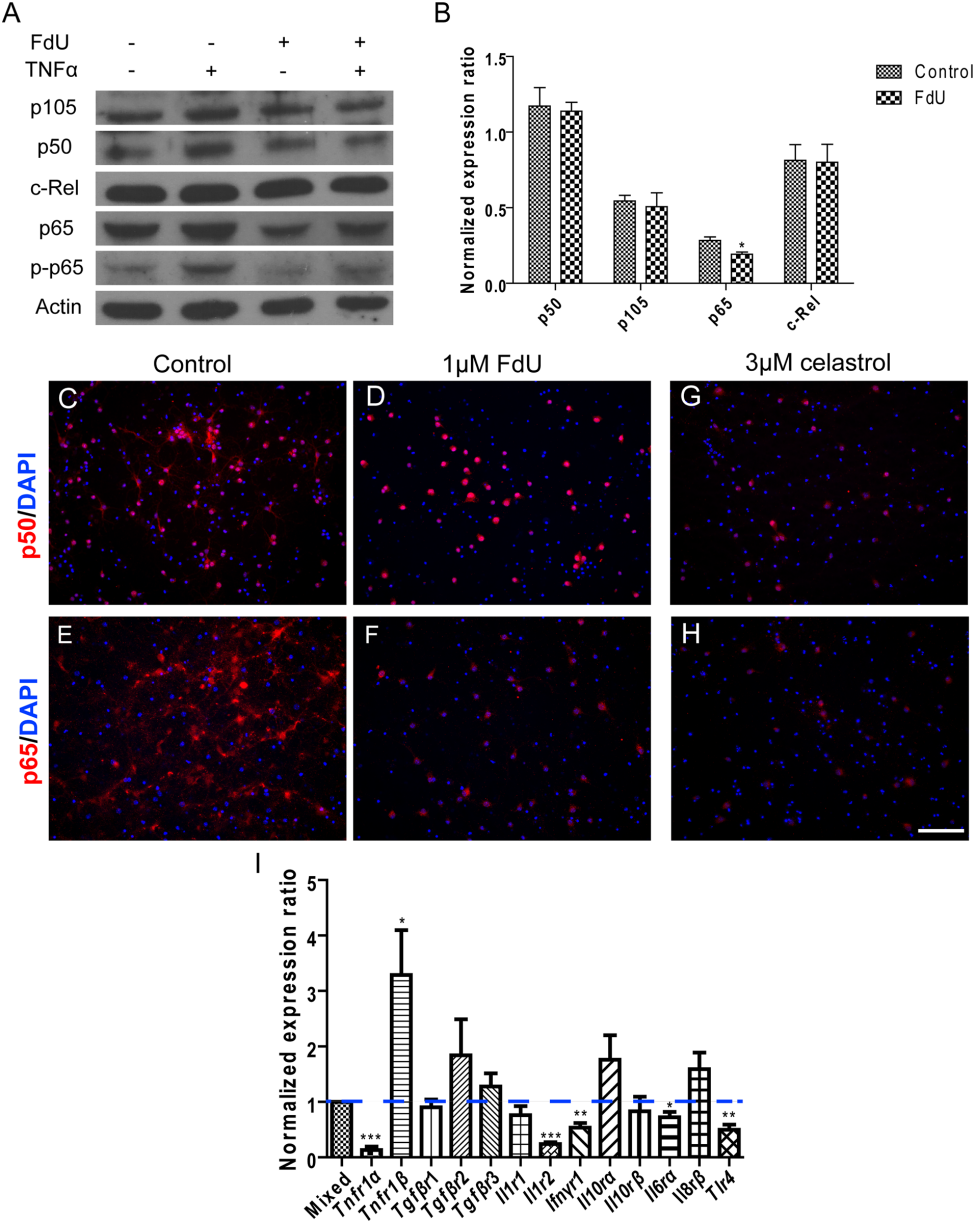
Differential expression of NFκB subunits and cytokine receptor genes in mixed and FdU-treated neuronal cultures. (A) Western blot of various NFκB subunits as indicated in control (Lanes 1,2) and FdU-treated (Lanes 3.4). TNFα treatment (Lanes 2,4) was used to measure the inflammatory response of the cultured cells. See text for details. (B) Quantification of the Western results shown in Panel A. (C-H) Immunostaining for p50 (C,D,G) and p65 (E,F,H) in FdU-treated (D,F) and untreated (C,E) cultures. (G-H) The NFκB antagonist, celastrol, confirmed the specificity of NFκB signals. Scale bar = 50μm. *: *p*< 0.05, **: *p*< 0.01, ***: *p*< 0.001. n = 3 for each group. (I) Q-PCR values of transcripts of various cytokine receptors (X-axis) in FdU-treated cultures are shown as ratios of the values found in untreated cultures.

NFκB plays a central role in the inflammation signaling pathway and helps to establish the proinflammatory environment of microglial and astrocytic activation [19, 23, 24]. We therefore determined the levels of NFκB subunits in mixed and FdU-treated cultures. We found that NFκB2/p52 and RelB were virtually absent (data not shown) while NFκB1/p50, p105 (the precursor of p50), RelA/p65 and c-Rel were present in both cultures (Fig 5A). The level of p65 was slightly but significantly reduced in FdU-treated cultures (Fig 5B). This suggests that p50 and c-Rel are mainly neuronal NFκB subunits, while p65 is present in both neurons and non-neuronal cells. We next determined NFκB responsiveness by exposing the cultures to TNFα for 1 hour. We used p65 phosphorylation as a measure of NFκB activity and found that its levels were elevated by TNFα in both mixed and FdU-treated cultures. The responsiveness of the neuron-enriched cultures however, was considerably diminished (Fig 5A). The significance of this data was extended with p50 and p65 immunostaining. As predicted by the Western blots (Fig 5A and 5B) p65 localized in both neurons and glial cells. Therefore, the removal of glial cells results in a significant loss of p65 protein staining (Fig 5C–5F). To confirm the specificity of signals, the NFκB inhibitor, celastrol, was added into the cultures for 24 hours. This greatly reduced both p50 and p65 immunostaining (Fig 5G and 5H) as well as the intensity of their respective bands on Western blots (data not shown).

To determine the cytokine responsiveness of the cells in our cultures, we performed reverse transcription and qRT-PCR to monitor mRNA levels for the different cytokine receptors in mixed versus FdU-treated cultures. Most receptor message levels were not significantly different in FdU-treated and untreated cultures. For five receptors, however, we found a significant decrease in the neuron-enriched cultures. These include the apoptosis-promoting TNFR1α, an IL1 decoy receptor (Il1r2), the interferon-γ receptor, the IL6rα receptor and *Tlr4* (the LPS receptor). All were significantly reduced in pure neuronal culture (Fig 5I). Only one receptor was significantly increased in the FdU-treated cultures—the apoptosis-preventing TNFα receptor, TNFr1β (Fig 5I). We would suggest that the receptors whose levels are reduced are predominantly expressed in non-neuronal cells or require the non-neuronal cells to stimulate expression in neuronal cells. Their absence offers a potential explanation for the loss of immune responsiveness (e.g., to LPS stimulation) in the FdU-treated cultures.

### Differential responses to individual cytokines and THP-1 conditioned medium between mixed and pure neuronal culture

**Fig 6:**
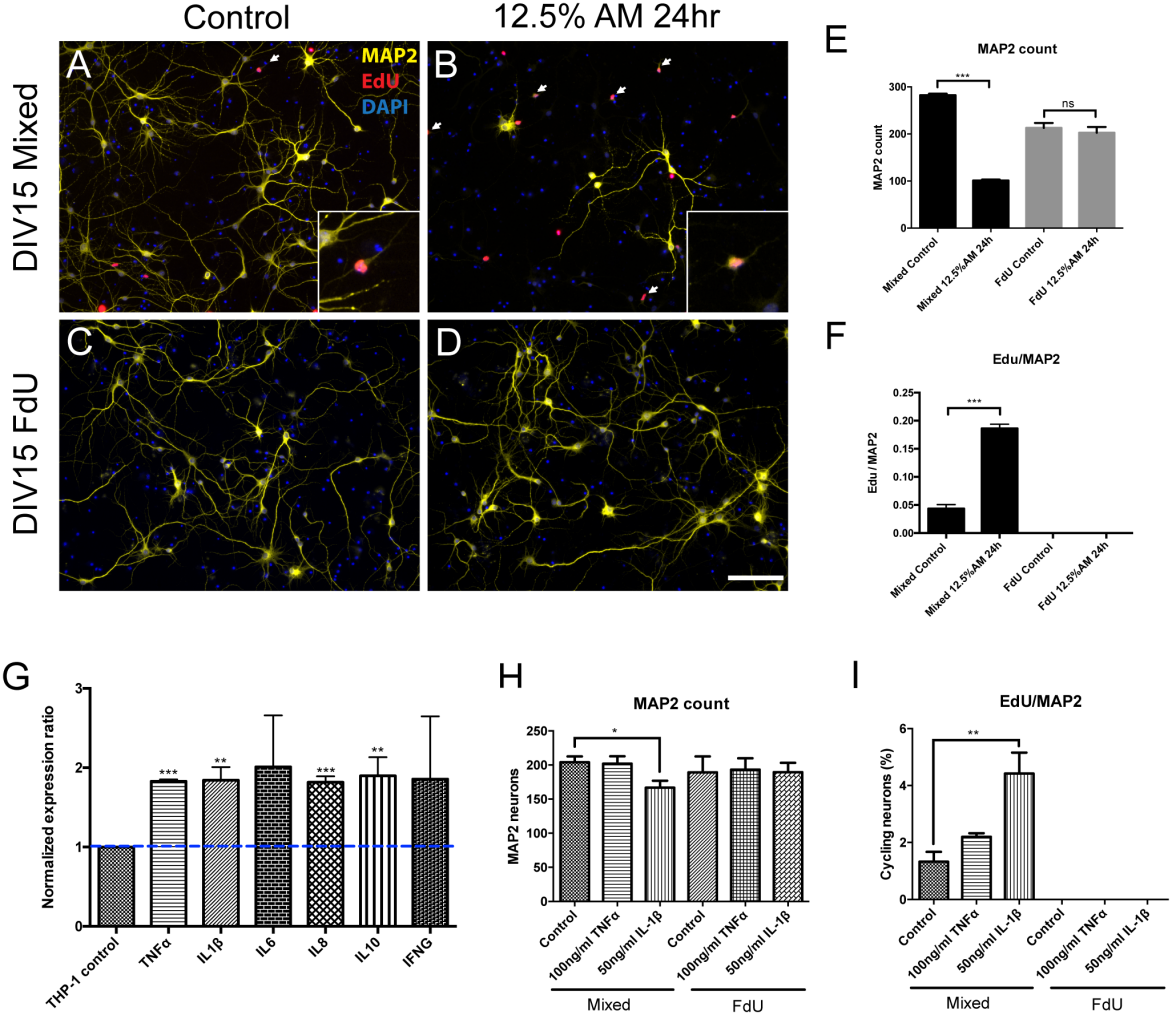
Inflammation induced neuronal damage requires the presence of non-neuronal cells. Addition of 12.5% THP-1 amyloid conditional medium (AM) triggered both neuronal death and cell cycle reentry in mixed culture (A-B) but failed to do so in FdU-treated culture (C-D). Quantification of Map2 cell numbers (E) and neuronal cell cycle activity (F) confirmed these qualitative observations. (G) AM induced cytokine gene expression in mixed culture as measured by qPCR (all values relative to THP-1 control). Application of individual pro-inflammatory cytokines validated the findings with AM treatment, both with respect to neuronal cell numbers (H) and cell cycle events (I). Scale bar = 50 μm. *: *p* < 0.05, **: *p* < 0.01, ***: *p* < 0.001. n = 3 for each group.

We have previously shown that soluble factors released by β-amyloid activated mouse brain microglia or THP-1 monocytes induce a cell cycle related death in mouse cortical neuronal cultures [15]. To investigate the contribution of other glia to this effect, we treated both mixed and FdU-treated neuronal culture with conditioned medium from THP-1 cells that had been stimulated by Aβ_1–42_ (AM—amyloid conditioned medium). Consistent with our previous findings, AM treatment caused significant neuronal death as well as cell cycle reentry in mixed cortical culture (Fig 6A, 6B, 6E and 6F). Unexpectedly, however, these effects were completely abolished in FdU-treated cultures (Fig 6C, 6D, 6E and 6F), suggesting that cell cycle related neurodegeneration induced by AM was dependent on the presence of glial cells. That the stimulated THP-1 cells were indeed producing cytokines after Aβ_1–42_ stimulation was confirmed by RT-qPCR. Expression of both pro- and anti-inflammatory cytokines and chemokines (IL1β, TNFα, IL8 and IL10) were all elevated (Fig 6G).

We next treated both types of cultures with individual cytokines—TNFα and IL1β. Consistent with our previous findings [25], TNFα induced neither neuronal death nor cell cycle reentry, while IL1β triggered both a 4-fold increase cell cycle events and a 20% loss of MAP2-postiive cells. Consistent with the results of the AM exposure, however, in FdU-treated cultures, IL1β treatment produced no significant loss of MAP2-positive cells and was unable to trigger any neuronal cell cycle activity (Fig 6H and 6I). Taken together, these data suggest that non-neuronal cells are necessary to mediate the effects of inflammatory challenges, such as exposure to LPS, THP-1 conditioned medium and IL1β in traditional cultures of embryonic mouse cortex.

## Discussion

Primary neuronal cultures are widely used to study neuronal activity at the cellular and molecular levels. As embryonic neural precursor cells can divide and differentiate into neurons or glial cells, primary neuronal cultures are usually regarded as mixed—containing both nerve cells and glia [1]. To investigate individual activities of neurons or glia, and the interaction between them, enrichment of the different constituent cell types is needed. Arabinosylcytosine C (AraC) and 5-Fluoro-2’-deoxyuridine (FdU) are two anti-mitotic reagents that have been used to remove proliferating cells and therefore obtain pure neuronal cultures. We choose FdU rather than AraC to enrich our cultures for neurons as no neuronal damage is reported after FdU treatment. In a previous study, at least 5 μM FdU was applied to the cortical cultures and kept in the medium for several days [6, 7, 10, 26]. Neuronal cultures were usually harvested before DIV10, so the status of mature neuron (DIV13-21) in pure cortical neuronal culture was unclear. Given the fact that mature neuronal culture is preferred for the study on neurodegenerative diseases, especially Alzheimer’s, we checked whether these traditional protocols were suitable for the purification of mature neurons in longer term cultures. We found that neurons in cultures treated with 5 μM or more FdU from DIV4 until DIV15, underwent severe damage to their structure and significant loss of numbers. This led us to seek to improve the method to be appropriate for longer-term experiments.

We find that a 24 hour treatment of low concentrations of FdU is sufficient to eliminate most dividing cells and to block further cell proliferation in neuronal cultures even 2½ weeks after FdU exposure. The loss of non-neuronal cells was documented by the loss of protein markers for astrocytes (GFAP) and OPCs (NG2) in FdU treated cultures. We also checked for microglia (Iba1 immunostaining) which are largely absent in our mixed and FdU cultures. Equally important, we provide evidence that the modified protocol allows neurons to survive, to mature and to remain relatively healthy. We find a robust presence of synaptic structures in both pure and mixed cultures. The excitotoxicity response of the FdU-treated cultures to L-glutamate is additional evidence that the constituent neurons retain at least basic synaptic function.

The ability to greatly reduce the representation of glial cells in our in vitro cultures provides a valuable model system in which to study the interaction between the neuronal and non-neuronal cells of the brain. This has great significance, as it is the interactions between glia and neurons that are increasingly recognized as critical for the initiation or progression of a wide range of neuropathological conditions [27–29]. For example, chronic inflammation is known to play an important role in the pathogenesis of neurodegenerative diseases, especially in Alzheimer’s [30–32]. In support of this concept, previous studies from our lab have demonstrated that Aβ activated primary microglia or THP-1 monocytes induce cell cycle related neuronal death[15, 33]. Chronic inflammation is also observed in traumatic brain injury (TBI). The primary injury in TBI is mechanical and results from shearing, tearing, and/or stretching of neurons, axons, glia, and blood vessels. The primary injury triggers secondary events such as the long-term activation of inflammation and prolonged activation of microglia. This leads in turn to the excessive production of pro-inflammatory cytokines, excitotoxicity, oxidative stress, mitochondrial dysfunction, blood—brain barrier (BBB) disruption, progressive neurodegeneration, downregulation of neurogenesis in hippocampus and delayed cell death [34–36]. While it might seem in cell culture models that the neurotoxic effects of inflammatory reactions were direct on the neurons themselves, when we used our neuron-enriched model system, we discovered an obligate role for the non-neuronal cells in the response of the neurons to multiple inflammatory challenges.

Similarly, we had hypothesized that diverse inflammatory factors released by microglia or THP-1 monocytes induced cell cycle related neuronal death by interacting directly with the neurons in our cultures. Yet we find that in FdU-treated cultures, in the absence of most glial cells, cultured cortical neurons show very different responses to AM and to IL1β treatment. FdU-treated DIV14 cultures of E16.5 mouse cortical neurons cannot be induced either to re-enter a cell cycle or to die after exposure to IL1β or the cytokine mixture found in AM. These results virtually eliminate a model where the impact of the cytokines is felt directly by the neurons.

To explore this finding more fully we examined the NFκB signaling pathway that plays a central role in the neuroinflammatory response during the pathogenesis of neurodegenerative disorders [37–39]. Typically, NFκB activation requires phosphorylation and degradation of the inhibitory molecule IκBα. This releases the p50-p65 heterodimer, allowing it to translocate to the nucleus. We found significant levels of NFκB1/p50 and c-Rel in neurons (they were found in both FdU-treated and untreated cultures), but RelA/p65 was found in both, although predominantly in glia (present in normal cultures, but greatly reduced in FdU-treated ones). The phosphorylation of p65 at Ser276 by protein kinase A (PKA) is thought to facilitate p65 DNA binding, while phosphorylation at Ser536 enhances its transactivation potential [40–42]. Indeed, phosphorylation of p65 often serves as a marker of NFKB activity [43, 44]. The near total absence of p65 from the FdU-treated cultures suggests that neurons depart from the traditional NFκB signaling pathway. One possible explanation of these results is that the down-regulation of NFκB activity protects neurons against inflammatory stresses such as THP-1 conditioned medium, LPS or IL1β.

Expression of several pro-inflammatory cytokine receptor genes, including *Tlr4*, *Tnfr1α*, *Il6rα* and *Ifnγr1*, were significantly decreased in FdU-treated cultures, which helps explain the reduced neuronal cytokine responses [33, 45–47]. At the same time, gene expression of TNFR1β was dramatically increased in the neuron-enriched cultures. It is noteworthy that TNFR1β has been proposed to block TNFα induced apoptosis due to the lack of a death domain in its structure [48]. We noted that the neuronal response to IL1β, though negative, was probably attenuated after glia depletion. This may be explained in part by our finding that expression of the decoy receptor gene *Il1r2* was greatly reduced in FdU-treated cultures, while the expression of *Il1r1* trended lower. The neuronal resistance to direct IL1β exposure is consistent with previous findings of glial-mediated reductions in IL1β-induced neurotoxicity [49].

How the non-neuronal cells mediate the inflammation-induced neurodegeneration in our normal cultures is still far from clear. Perhaps, as has been suggested, pro-inflammatory cytokines amplify neuronal damage by causing astrocytes to flood their associated neurons with NO [50]. It is also possible that astroglial NFκB activation leads to the release of complement factor C3 a protein with a known association with AD [38]. Finally, the possibility that physical glia-neuron contact is required for inflammation-mediated neurodegeneration must also be considered.

In summary, we report a modified FdU-treatment protocol for the enrichment of neurons in cultures of embryonic mouse cortex. The abbreviated exposure that we have developed is effective at removing most of the mitotic non-neuronal elements while allowing the neurons maintain a healthy morphology and function. We have demonstrated the value of the model by exploring the response of the cultures to different neuroinflammatory challenges. Based on our results, it appears that that the non-neuronal cells of a typical cortical culture are required to mediate most if not all of the degenerative phenotypes induced by inflammation. The current study will be extended to in our Alzheimer's and Ataxia animal models in order to define whether neuronal or glial cells are the primary mediators of the diseases and thus provide important new insights into preventive and therapeutic treatments.
